# Three-Dimensional Changes in the Mandibular Proximal Segment After Using a Surgery-First Approach in Patients With Class III Malocclusion and Facial Asymmetry

**DOI:** 10.1097/SCS.0000000000008520

**Published:** 2022-02-16

**Authors:** MyungSu Kim, Nayansi Jha, Jae-Hong Choi, Yoon-Ji Kim, Uilyong Lee, Lucia Cevidanes, Jin-Young Choi, Seung-Hak Baek

**Affiliations:** * Department of Orthodontics, Korea University Graduate School of Dentistry, University of Ulsan, South Korea;; † Graduate School of Medicine, University of Ulsan, South Korea;; ‡ Department of Orthodontics, Asan Medical Center, College of Medicine, University of Ulsan, South Korea;; § Department of Oral and Maxillofacial Surgery, Chungang University Hospital, Seoul, South Korea;; ∥ Department of Orthodontics and Pediatric Dentistry, University of Michigan School of Dentistry, Ann Arbor, MI;; ¶ Department of Oral and Maxillofacial Surgery, Seoul National University, Seoul, South Korea.; # Department of Orthodontics, School of Dentistry, Dental Research Institute, Seoul National University, Seoul, South Korea.

**Keywords:** Facial asymmetry, jaw surgery, malocclusion, orthognathic surgery, sagittal split ramus osteotomy

## Abstract

This study was performed to evaluate condylar position and angulation after asymmetric mandibular setback between a conventional (CA) and surgery-first approach (SFA) using three-dimensional analysis. The condylar positions of 30 patients with skeletal Class III malocclusion and facial asymmetry who underwent 1-jaw (sagittal split ramus osteotomy) or 2-jaw orthognathic surgery (Le Fort I osteotomy and sagittal split ramus osteotomy) with CA (n = 18) or SFA (n = 12) from 2 university hospitals were studied. The three-dimensional assessment of condylar changes was performed using computed tomography images at the initial time point (T0) and at least 6 months after surgery (T1). Segmentation of condyles and cranial base assessment from cone-beam computed tomography images were performed using ITK-SNAP software (version 3.4.0). Condylar position and angulation changes were calculated using 3D Slicer software (version 4.10.2), and statistical analysis was performed. No significant translational or rotational condylar changes were observed between the deviated and nondeviated sides in each group or between the CA and SFA groups except yaw (*P* = 0.014). Linear mixed-model analysis and multivariate analysis showed no significant difference between the CA and SFA groups. Surgery-first approach might not be associated with more harmful effects on the condylar position and angulation changes as compared with CA.

There are several advantages to using the surgery-first approach (SFA) for orthognathic surgery, including immediate improvement in facial esthetics, shorter treatment time, and enhanced patient satisfaction and quality of life, as compared with the conventional approach (CA); moreover, the SFA has a stable surgical outcome similar to that of CA.^1–9^ Patients with skeletal Class III malocclusion and facial asymmetry usually show extrusion of the maxillary second molars because of the absence of occlusal contact with the opposing mandibular second molars. In patients with facial asymmetry, lateral compensation of the canines and the posterior teeth are observed.^10^ However, when using surgical correction for facial asymmetry, there may be a higher tendency for relapse because SFA is associated with greater postsurgical changes in occlusion.^11^

Condylar displacement that occurs during surgery depends on various factors, including the surgeon’s experience, the bony interferences between the proximal and distal segments, type of fixation, and so forth.^12^ The mandibular setback procedure using sagittal split ramus osteotomy (SSRO) pushes the proximal segment of the mandible backward. However, the action of the masseter and temporalis muscles makes it rotate counterclockwise. Therefore, accurate positioning of the condyle of the mandible after SSRO is known to be a major factor for surgical stability.^12–16^ Because of the unstable surgical occlusion associated with SFA as compared with CA, SFA might result in a greater amount of condylar displacement. In addition, several previous studies have been performed to analyze the amount and direction of condylar displacement.^12,17–21^ Although efforts have been made to control the position of the proximal segment to ensure surgical stability,^15,22^ these previous studies mostly assessed the condylar positional changes in two-dimensional (2D) radiographs such as lateral cephalograms and 2D reformatted images derived from the computed tomography (CT). Soverina et al,^9^ in their systematic review, reported that because all articles described stability using a penultimate time point of “after surgery” and not “after debonding,” orthodontic movements and consequent mandibular movements could have influenced cephalometric measurements. Therefore, to verify the real stability of the SFA, it is necessary to perform further studies with longer follow-up periods and evaluation at the same time points.^9^

The purpose of this study was to compare the amount of change in the condylar position and angulation after asymmetric mandibular setback between CA and SFA using three-dimensional (3D) analysis with long-term follow-up. The null hypotheses were as follows:

1. there is no difference in the amount of change in the condylar position and angulation changes between the CA and SFA patients, and
2. there is no difference in the pattern of condylar displacement between the deviated and nondeviated sides.

## MATERIALS AND METHODS

### Subjects

This retrospective multicenter study was approved by the institutional review board of Seoul National University Dental Hospital (ERI20022) and Korea University Anam Hospital (2019AN0011). Subjects consisted of patients who underwent orthognathic surgery with either CA or SFA. Inclusion criteria were as follows:

1. patients who had skeletal Class III malocclusion and facial asymmetry (menton deviation, >3 mm; measured from the midsagittal reference plane constructed by the crista galli, anterior nasal spine, and opisthion),
2. patients who had undergone either 1-jaw or 2-jaw orthognathic surgery,
3. patients whose mandible was surgically moved backward through the use of a modified SSRO with short lingual osteotomy design, and
4. patients who underwent CT before (T0) and at least 6 months after surgery (T1).

The exclusion criteria were as follows:

1. patients who had cleft lip and palate or craniofacial syndromes,
2. patients who had a history of previous jaw surgery,
3. patients who had signs and symptoms related to the temporomandibular joint arthritis, and
4. patients who had medically compromised conditions.

All patients had semi-rigid fixation using metal plates. After surgery, no maxillomandibular fixation was performed and intermaxillary elastics were worn for 3 to 4 weeks.

Thirty patients were ultimately selected and divided into 2 groups: the CA group (n = 18; mean age, 19.8 years; mean menton deviation, 5.2 mm; 4 one-jaw and 14 two-jaw surgery cases; mean follow-up duration, 14.3 ± 9.1 months; Supplementary Digital Content, Table 1, http://links.lww.com/SCS/D799) and the SFA group (n = 12; mean age, 21.3 years; meanmenton deviation, 4.9 mm; 2 one-jaw and 10 two-jaw surgery cases; mean follow-up duration, 17.3 ± 7.7 months, Supplementary Digital Content, Table 1, http://links.lww.com/SCS/D799).

### Data Acquisition

Computed tomography (CT) or cone-beam CT (CBCT) images were taken 1 month before (T0) and at least 6 months after surgery (T1). At Seoul National University Dental Hospital, multispiral CT (Sensation 10, Siemens, München, Germany; axial slice thickness, 1.0 mm) was used. At the Korea University Hospital, CBCT (Kavo Dental GmBH, Biberach, Germany) was used.

### Creation of the Condylar Models

**FIGURE 1.**
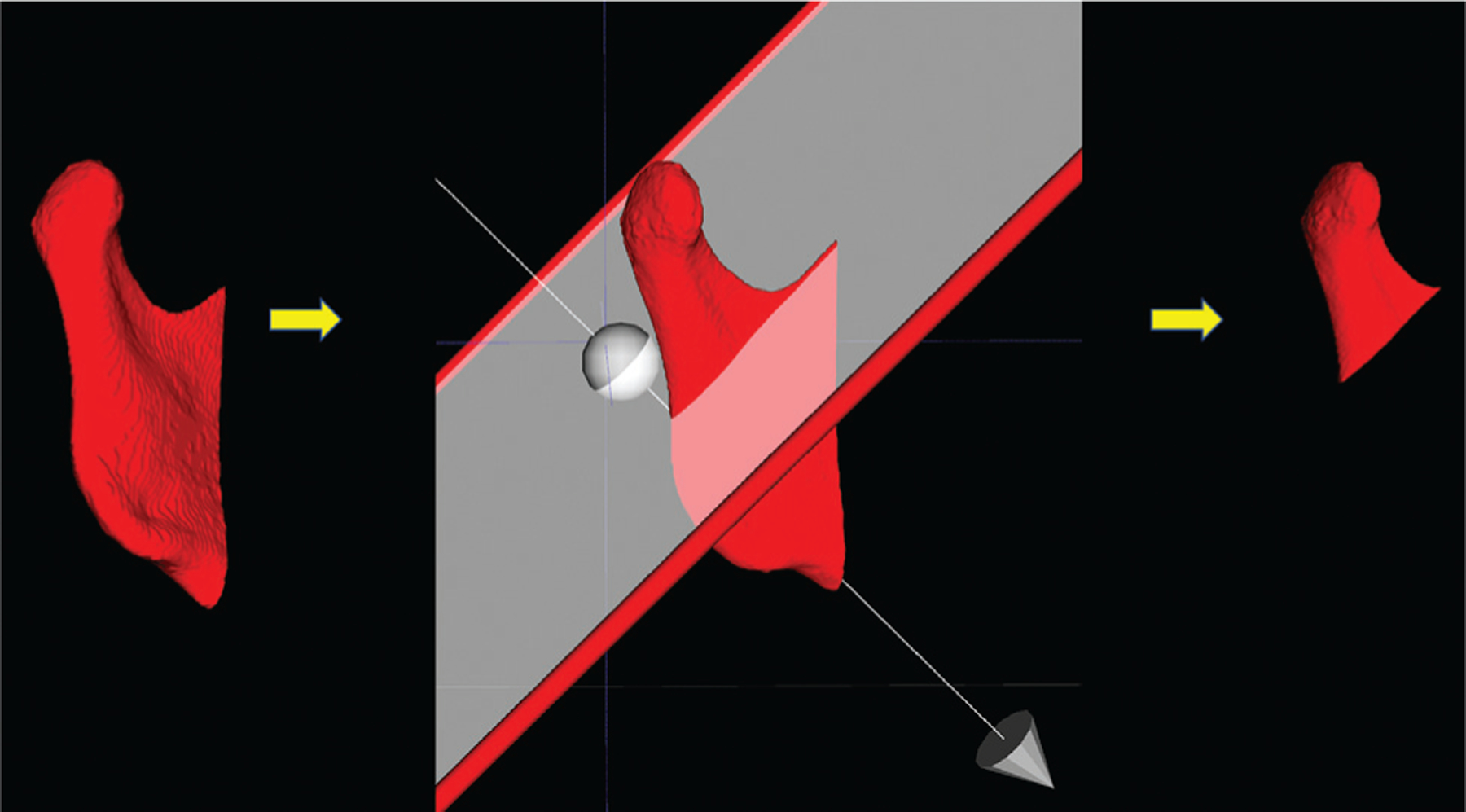
Segmentation of the condyles of the multispiral computed tomography (CT) and cone-beam computed tomography (CBCT) images at the T0 and T1 stages performed using the ITK-SNAP software. Segmented condyles were cut using the scalpel tool (ITK-SNAP software) to obtain condylar head for further assessment.

**FIGURE 2.**
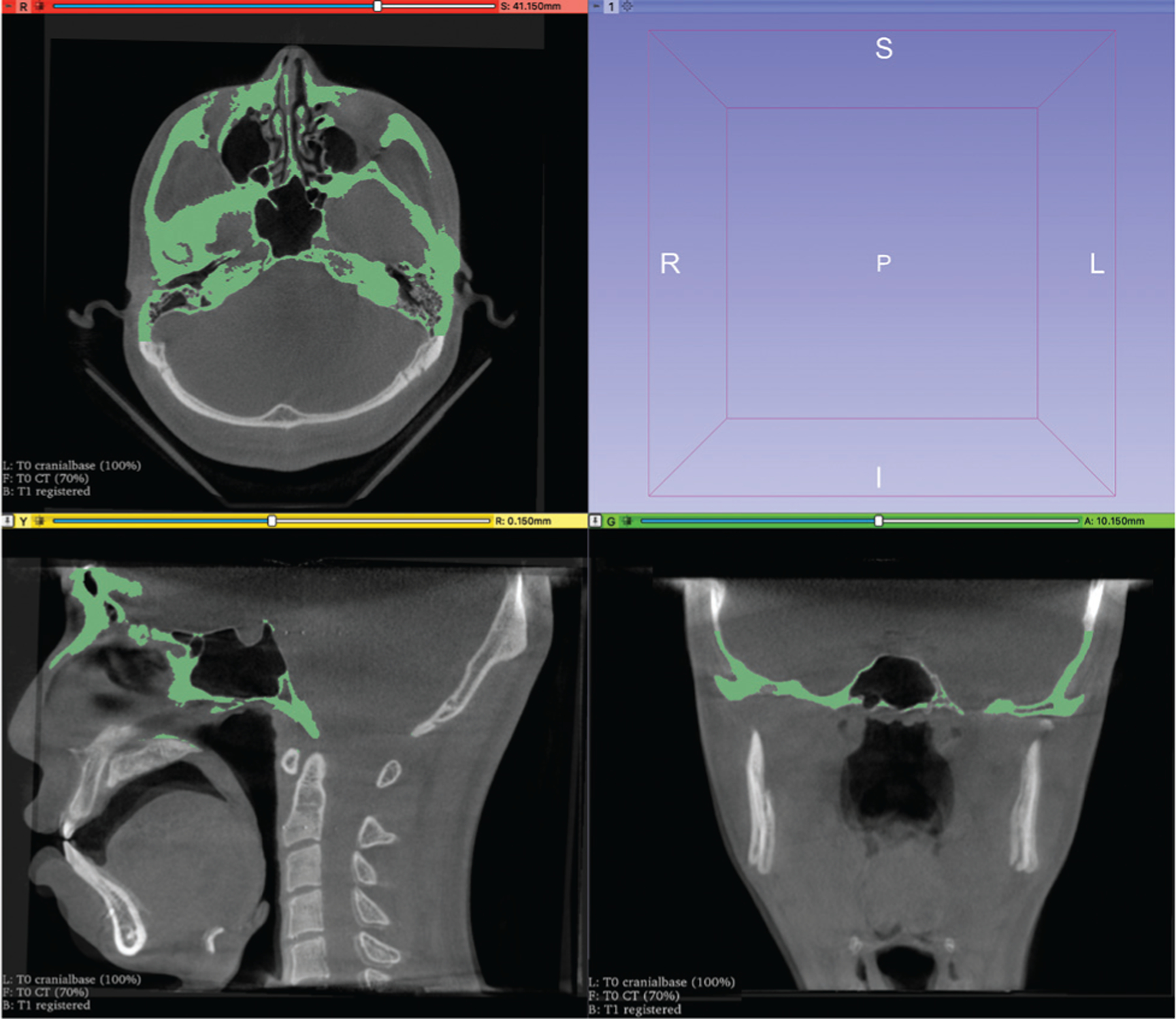
Voxel based registration with cranial base as reference, using 3D Slicer. The condyle at the T0 stage was registered with that of the T1 stage using the cranial base as a reference. 3D, three-dimensional.

For the 3D quantitative assessment of condylar displacement after orthognathic surgery, segmentation of the condyles of the multispiral CT and CBCT images at the T0 and T1 stages were performed using ITK-SNAP software (open source, version 3.4.0; http://www.itksnap.org)^23^ (Fig. 1). The condyle at the T0 stage was registered with that of the T1 stage using the cranial base as a reference (Fig. 2).

### Landmarks and Measurements of Variables

**FIGURE 3.**
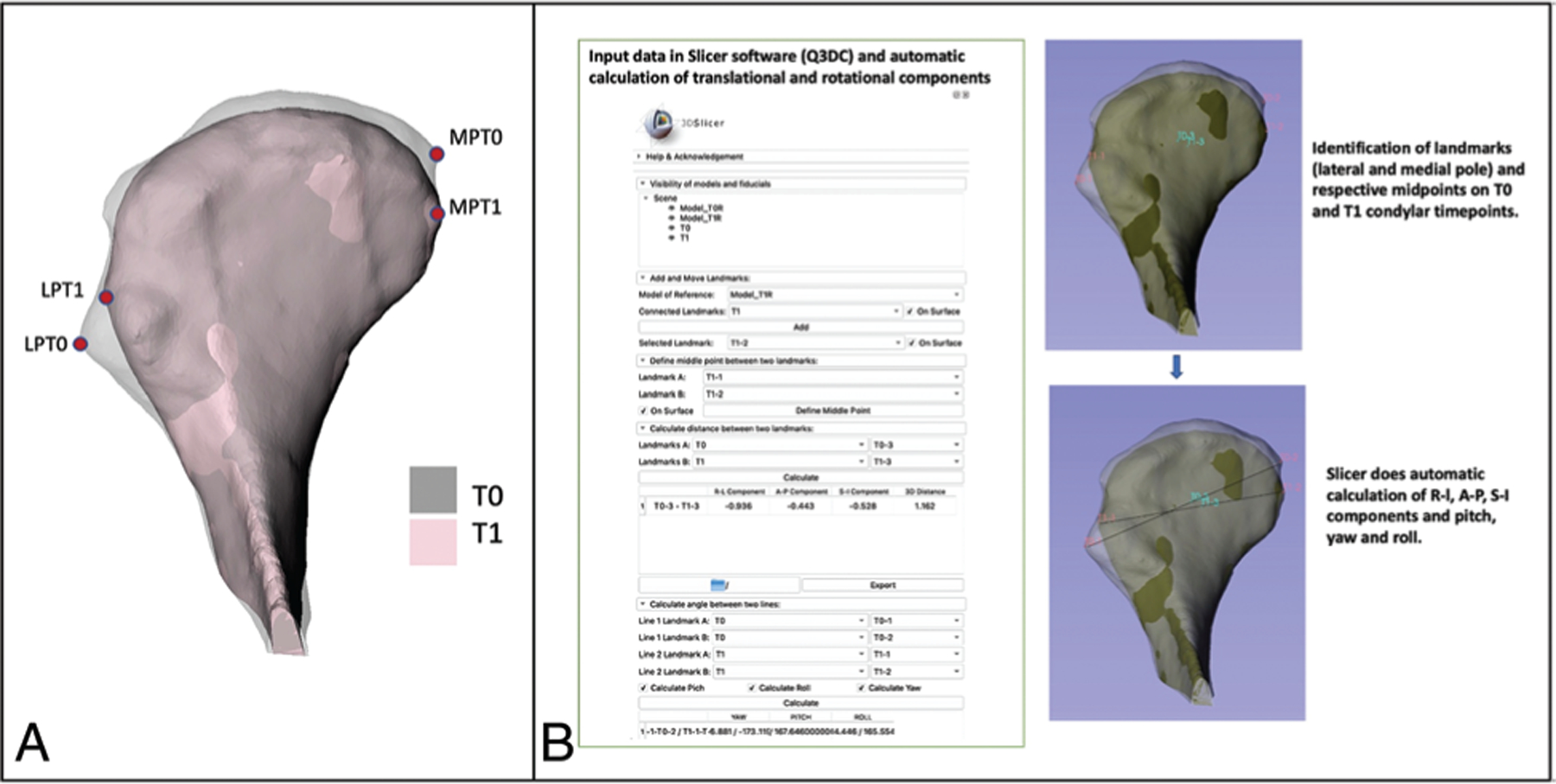
(A) Landmark positioning was done on 4 points on the condylar head (2 points on medial pole and 2 points on lateral pole), and Q3DC module of the 3D Slicer software calculated the translational and rotational displacements. (B) Translational and rotational changes of the condyles were measured using the Q3DC module of the 3D Slicer software. LP, lateral point; MP, medial point; Q3DC, quantitative 3D cephalometrics; T0, image before surgery; T1, image after surgery.

**FIGURE 4.**
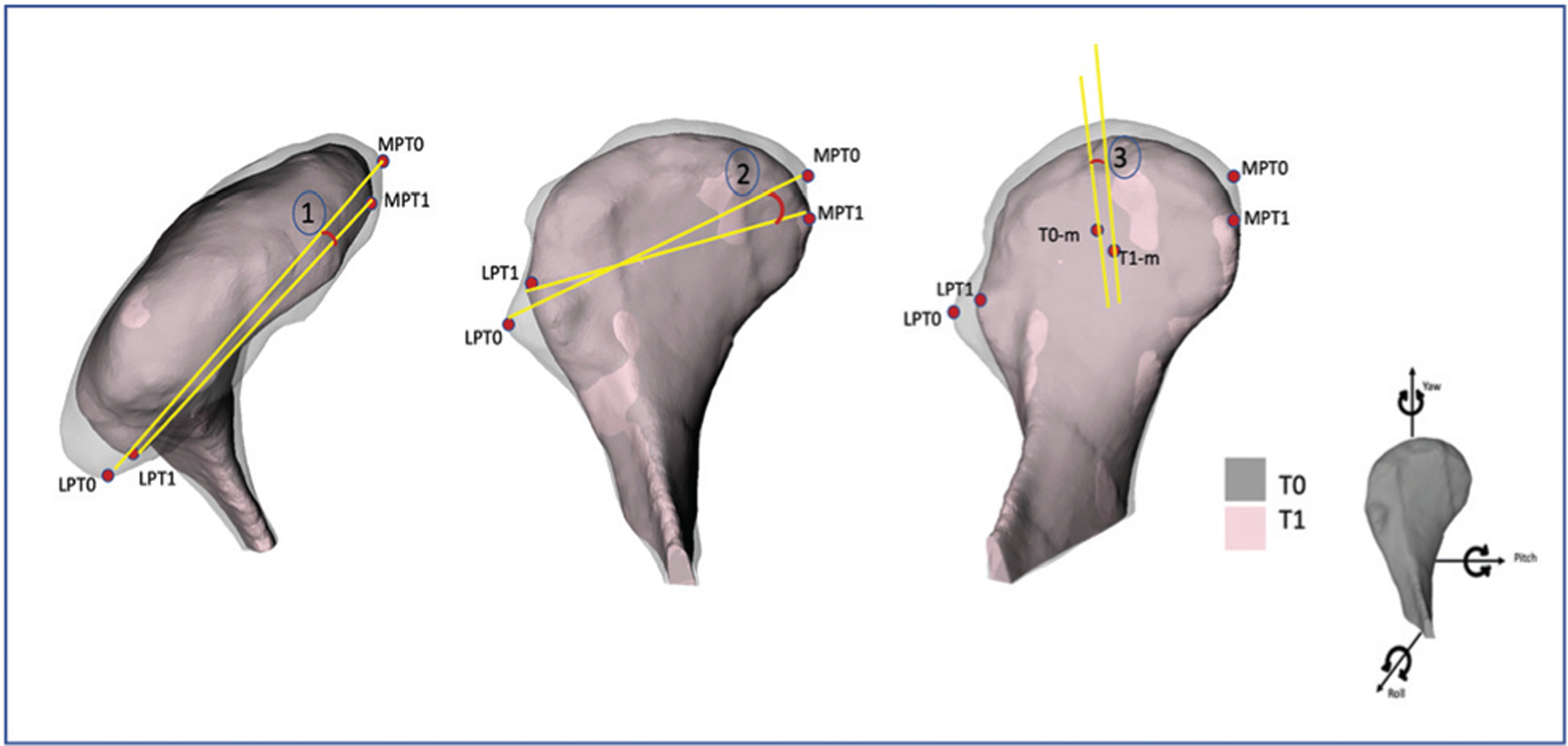
Rotational condylar changes with SLICER software. 1: yaw (axial view), 2: roll (coronal view), 3: pitch (sagittal view). LP, lateral point; MP, medial point; T0, image before surgery; T1, image after surgery; T0 m, midpoint between LPT0 and MPT0; T1 m, midpoint between LPT1 and MPT1.

Landmark positioning was performed on the condylar heads. A total of 4 points were marked, 1 point on the medial pole and 1 point on the lateral pole of the condylar head for both T0 and T1 time points (Fig. 3A). Translational and rotational changes of the condyles were measured using the quantitative 3D cephalometrics module of the 3D Slicer software (open source, version 4.10.2; http://www.slicer.org),^24^ which allows direct measurement of the condylar displacement in the 3D models (Fig. 3B). After the medial and lateral poles of the condylar head were marked, the software automatically calculated the translational displacement in the sagittal (forward or backward), vertical (upward or downward), and transverse (right or left) directions as well as the rotational changes in the coronal (roll), axial (yaw), and sagittal planes (pitch) (Fig. 4).

### Validation of Variable Measurement

Segmentation and measurement of the condylar displacement of all multispiral CT and CBCT images was performed by the same operator (MSK) at 4-week intervals. In terms of intraexaminer reproducibility, the intraclass correlation coefficient values for the linear measurements (sagittal, vertical, transverse) ranged from 0.86 to 0.91, and the angular measurements (yaw, pitch, roll) ranged from 0.82 to 0.99 (data not shown), which indicated excellent reproducibility. The Dahlberg error ranged from 0.17 to 0.27 for linear measurements and from 2.6 to 7.9 for angular measurements. We used the first set of measurements for further analysis.

### Statistical Analysis

For the statistical analysis, we performed Fisher exact test, Mann–Whitney *U* test, Wilcoxon signed-rank test, and linear mixed-model analysis using SPSS statistical software, version 22 (SPSS Inc, Chicago, IL). A *P* value of less than.05 was considered statistically significant.

## RESULTS

### Comparison of Demographic Data Between the 2 Groups

The mean operation age, sex, mean menton deviation, distribution of 1-jaw surgery and 2-jaw surgery, mean duration of follow-up, and amount of mandibular setback in the greater (nondeviated) and lesser setback (deviated) side did not differ between the CA and SFA groups (19.8 versus 21.3 years; 8 males and 10 females versus 8 males and 4 females; 5.2 versus 4.9 mm; 4 one-jaw and 14 two-jaw surgery cases versus 2 one-jaw and 10 two-jaw surgery cases; 14.3 ± 9.1 versus 17.3 ± 7.7 months; *P* > 0.05; Supplementary Digital Content, Table 1, http://links.lww.com/SCS/D799).

### Comparison of Amount of Translational and Rotational Changes of the Condyle of the Mandible During T0–T1 Between the Deviated and Nondeviated Sides in Each Group

There was no significant difference in the translational displacement of the deviated and nondeviated sides in the CA group (transverse: −0.14 versus 0.11 mm; sagittal, −0.06 versus 0.06 mm; vertical, 0.04 versus −0.19 mm; all *P* > 0.05). There was also no significant difference in the angular change between the deviated and nondeviated sides, except for yaw (yaw, 2.77° versus 0.14°, *P* = 0.014; pitch, 2.06° versus 2.2°, *P* > 0.05; roll, −1.41° versus 0.41°, *P* > 0.05; Supplementary Digital Content, Table 2, http://links.lww.com/SCS/D799).

In the SFA group, there was no significant difference in the translational displacement of the deviated and nondeviated sides (transverse: 0.19 versus −0.11 mm, *P* > 0.05; sagittal, −0.33 versus 0.21 mm, *P* > 0.05; vertical, 0.2 versus −0.36 mm; *P* > 0.05). There was no significant difference in the angular change between the deviated and nondeviated sides (yaw, 2.15° versus 0.05°, *P*> 0.05; pitch, −0.22° versus 3.32°, *P* > 0.05; roll, −0.08° versus −0.62°, *P*> 0.05).

### Comparison of the Amount of Translational Changes of the Condyle of the Mandible During T0–T1 Between the 2 Groups

In terms of translational change, there was no significant difference between the CA and SFA groups in the transverse, sagittal, and vertical displacement on the deviated side (−0.14 versus 0.19 mm; −0.06 versus −0.33 mm; 0.04 versus 0.2 mm; all *P* > 0.05) or on the nondeviated side (0.11 versus −0.11 mm; 0.06 versus 0.21 mm; −0.19 versus −0.36 mm; all *P* > 0.05; Supplementary Digital Content, Table 2, http://links.lww.com/SCS/D799).

### Comparison of the Amount of Rotational Changes of the Condyle of the Mandible During T0–T1 Between the 2 Groups

In terms of angular change, there was no significant difference in the yaw, pitch, or roll on the deviated side between the CA and SFA groups (2.77° versus 2.15°; 2.06° versus −0.22°; −1.41° versus −0.08°; all *P* > 0.05) or on the nondeviated side (0.14° versus 0.05°; 2.2° versus 3.32°; 0.41° versus −0.62°; all *P* > 0.05; Supplementary Digital Content, Table 2, http://links.lww.com/SCS/D799).

### Results of the Linear Mixed-Model Analysis and Multivariate Analysis

When controlling for the effects of age, sex, hospital (A versus B), orthognathic surgery type (1-jaw versus 2-jaw), and side (deviated side versus nondeviated side), no significant difference was observed between the CA and SFA groups (all *P* > 0.05; Supplementary Digital Content, Table 3, http://links.lww.com/SCS/D799). None of the independent variables and their interaction effects were significant for the condylar translational and rotational changes (all *P* > 0.05).

## DISCUSSION

### Findings of this Study

This study has some originalities, as follows

1. We evaluated the relatively long-term changes (mean duration, 15.56 months) in the condylar position and angulation in patients who had undergone orthognathic surgery with either CA or SFA.
2. Instead of using 2D reconstructed images, we used 3D anatomic landmarks, such as the medial and lateral poles of the condylar head, to directly measure condylar displacement.
3. Changes in the condylar position and angulation between deviated and nondeviated sides of the CA and SFA group did not yield any significant changes.

### Interpretation of Study Results

#### Positional Changes in the Condyle

**FIGURE 5.**
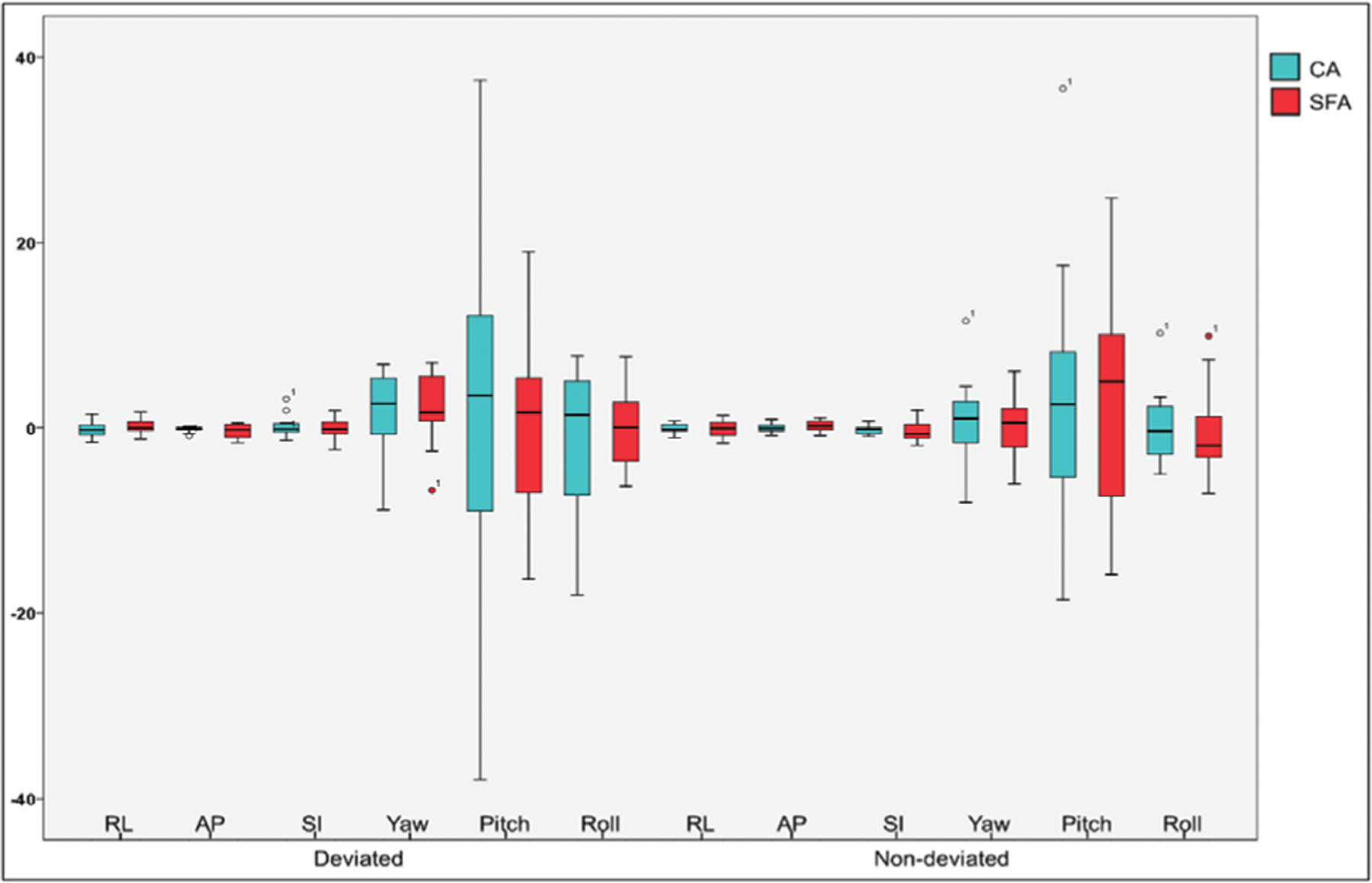
Boxplot of the amount of change in the condylar position and angulation in the deviated and nondeviated sides of the conventional (CA) and surgery-first approach (CFA).

Contrary to our expectations, there was no significant difference in the change in condylar position between the CA and SFA groups (Supplementary Digital Content, Table 2, http://links.lww.com/SCS/D799, Fig. 5). Our results are in line with the findings of a previous study by Jung et al,^21^ who used lateral cephalometric analysis to compare the changes in the proximal segment of the mandible in patients treated with SFA and CA. They showed similar positional changes between the 2 groups. Yang et al,^25^ also reported that although SFA requires a shorter duration of treatment than CA does, stability and surgical outcomes were similar between SFA and CA. In their lateral cephalometric analysis study of Class III patients who had undergone SSRO with SFA, Baek et al,^1^ reported that after the proximal segment rotated clockwise by 2° by SSRO, it moved forward to its pretreatment position during postsurgical orthodontic treatment. Because they observed an immediate forward movement of the mandible when the surgical wafer was removed, they suggested a possibility of clockwise rotation of the ramus during surgery.^1^

We observed the long-term follow-up (14 months after surgery) when the condylar position was stabilized. In their study on the condylar position of Class III patients treated with intraoral vertical ramus osteotomy (IVRO), Jung et al,^21^ showed that condylar displacement occurred immediately after surgery but was restored to the original position at 1 year after surgery. In our study, Class III patients were treated with SSRO and/or Le Fort I; however, we observed no significant changes between the deviated and nondeviated sides of the CA and SFA groups, except for the yaw rotation.

#### Rotational Changes in the Condyle

In the present study, there was no significant difference in the change in condylar angulation between the CA and SFA groups. In particular, the counterclockwise rotation (pitch) was approximately 2° to 3° for both the CA and SFA groups (Supplementary Digital Content, Table 2, http://links.lww.com/SCS/D799, Fig. 5). However, the standard deviation was large, which indicates high individual variability.

As the patients had prognathic mandible with asymmetry, differential setback was performed which resulted in the yaw movement of the distal segment by approximately 3° to 5°; there was no patient who underwent mandibular setback in the nondeviated side and advancement in the deviated side. In order to minimize the condylar displacement resulting from the yaw correction, a short lingual osteotomy was used. However, the yaw rotation of the condyle on the deviated side was observed for both CA and SFA groups, with no significant difference between the 2 groups (Supplementary Digital Content, Table 2, http://links.lww.com/SCS/D799). This result was similar to the findings of Kim et al,^26^ who reported the inward rotation of the condyle on the axial plane. In patients who had undergone IVRO, the yaw rotation of the condyle was also observed in both the CA and SFA groups without significant differences between the 2 approaches.^26^

However, the changes in angulation in the proximal segment were greater than the position changes on both the deviated and nondeviated sides, despite the lack of significant difference between the CA and SFA groups, respectively (Supplementary Digital Content, Table 2, http://links.lww.com/SCS/D799, Fig. 5).

For SFA patients, the mandibular setback procedure induces clockwise rotation and vertical opening of the mandible because of occlusal interferences, followed by counterclockwise rotation during postsurgical orthodontic treatment.^27^ However, immediately after surgery, the clockwise rotation of the proximal segment of the mandible of the SFA patients was not different from that of the CA patients. The proximal segment of the mandible is stable immediately after surgery because of the surrounding muscles, and after bone healing, the counterclockwise rotation of the proximal segment of the mandible may occur as the mandible is closed.

### Clinical Implications

In patients with skeletal Class III malocclusion and facial asymmetry, the amount of mandibular setback is different between the deviated and nondeviated sides. Thus, to minimize condylar displacement on the deviated side, surgical techniques have been developed to avoid the interferences between the proximal and distal segments.^26,28^ Yang et al,^29^ reported that modified SSRO with a short lingual osteotomy design showed the least displacement of the proximal segment, followed by IVRO and SSRO. Yang et al,^30^ suggested the use of the posterior bending osteotomy technique in Class III patients with facial asymmetry to minimize condylar displacement via a reduction in the contact between the proximal and distal segments.

In SSRO, the proximal segment is pushed backward by a mandibular setback procedure and rotates counterclockwise because of the action of the masseter and temporalis muscles. However, in IVRO, the proximal segment moves forward and downward with clockwise rotation.^31^ Because the amount of clockwise rotation is limited by the anatomy of the temporomandibular joint, IVRO might have a smaller net value of rotation as compared with that of SSRO.^31^ If greater range of rotation is observed, it is attributable to the change in the orientation of the masticatory muscles.^32^

Recent advances in 3D computer-aided design/computer-aided manufacturing technology has enabled clinicians to fabricate surgical wafers and simulate surgical movements^33^ as well as the validation of maxilla stability after orthognathic surgery.^34^ Simulation surgery and 3D computer-aided design/computer-aided manufacturing splints^35^ and condyle positioning jigs^15^ have been developed for such stabilization during orthognathic surgery. Lee et al,^22^ developed a type of computer-assisted surgery protocol for managing the proximal segment while treating complex maxilla–facial deformities.

### Study Limitations and Suggestions for Future Research

Because patients showed a large individual variability in condylar displacement (Supplementary Digital Content, Table 2, http://links.lww.com/SCS/D799), further studies are necessary with a prospective study design, large sample size, and stratification of subjects according to type of orthognathic surgery and direction and amount of surgical movement of the maxilla and mandible. In the present study, we determined condylar displacement using the medial and lateral poles of the condyle as anatomic landmarks. If significant condylar remodeling occurs during the follow-up period, errors in the assessment of the condylar position and angulation are inevitable. Therefore, the inclusion of more stable anatomic structures, such as the ramus, would lead to more reliable results. We obtained data from 2 different hospitals: one used CT, and the other used CBCT to obtain images, and this might have led to errors.

## CONCLUSIONS

The null hypotheses were rejected. Because there was no difference in the amount of changes in the condylar position and angulation between the deviated and nondeviated sides in each group and between the CA and SFA groups, SFA might not result in more harmful effects on condyle displacement as compared with CA.

## Supplementary Material

Supplementary material
