# Supplementary material for: Three-Dimensional Changes in the Mandibular Proximal Segment After Using a Surgery-First Approach in Patients With Class III Malocclusion and Facial Asymmetry
Source: J Craniofac Surg. Author manuscript; Available in PMC 2022 Oct 9. (PMC9378743; doi:10.1097/SCS.0000000000008520)
Supplement: Supplementary material [file NIHMS1793867-supplement-Supplementary_material.pdf]

Supplemental Table 1. Comparison of demographic data between the conventional approach (CA) and surgery-first approach (SFA)

	**CA (n = 18)**	**SFA (n = 12)**	***P* value**
Mean operation age, y[a]	19.8 ± 2.1	21.3 ± 2.3	0.104
Distribution of sex, M:F[b]	8:10	8:4	0.206
Mean menton deviation, mm[a]	5.2 ± 2.8	4.9 ± 2.1	0.760
Distribution of orthognathic surgery type, 1-jaw:2-jaw[b]	4:14	2:10	0.545
Mean time from the date of surgery to the follow-up date after surgery, mo[a]	14.3 ± 9.1	17.3 ± 7.7	0.305

[a] Mann–Whitney *U* test.

[b] Fisher exact test.

**Hospital**	**Orthognathic surgery**	**CA**	**SFA**	***P* value**
A	1-jaw	2	2	0.510
	2-jaw	3	6	
	Total	5	8	
B	1-jaw	2	0	0.574
	2-jaw	11	4	
	Total	13	4	

CA- conventional approach, SFA- surgery first approach

Supplemental Table 2. Comparison of the amount of the position and rotation changes of the proximal segment of the mandible during T0–T1 between the deviated and non-deviated sides in each group and between the conventional approach (CA) and surgery-first approach (SFA)[a]

		CA (n = 18)					SFA (n = 12)					Comparison of CA and SFA	
		**Deviated side**		**Non-deviated side**		***P* value**	**Deviated side**		**Non-deviated side**		***P* value**	**Deviated side**	**Non-deviated side**
		Mean	SD	Mean	SD		Mean	SD	Mean	SD		*P* value	*P* value
Position, mm	Δ Transverse	−0.14	0.76	0.11	0.54	0.152	0.19	0.82	−0.11	0.87	0.551	0.285	0.491
	Δ Sagittal	−0.06	0.35	0.06	0.46	0.339	−0.33	0.77	0.21	0.6	0.068	0.787	0.573
	Δ Vertical	0.04	1.04	−0.19	0.53	0.696	0.2	1.66	−0.36	1.08	0.378	0.884	0.518
Rotation, °	Δ Yaw	2.77	4.17	0.14	4.02	0.014	2.15	4.04	0.05	3.38	0.114	0.723	0.755
	Δ Pitch	2.06	18.29	2.2	12	0.628	−0.22	10.18	3.32	11.5	0.319	0.545	0.787
	Δ Roll	−1.41	6.79	0.41	3.56	0.673	−0.08	4.44	−0.62	5.11	0.630	0.819	0.325

[a] Mann–Whitney *U* test was performed. T0: pretreatment; T1: average 15.56 ± 8.6 months after surgery. A positive (+) sign indicates right, forward, and upward

Supplemental Table 3. Type III tests of fixed effects for the factors associated with long-term changes (T0 to T1) in the proximal segment of the mandible

**Independent variable**	***F***	***P***
Hospital (A vs B)	1.277	0.259
Group (CA vs SFA)	0.088	0.767
Sex	0.594	0.441
Surgery type (1-jaw vs 2-jaw)	0.422	0.516
Side (deviated vs non-deviated)	0.343	0.558
Movement type (displacement vs rotation)	2.145	0.144
Dimension	1.230	0.293
Age	0.193	0.661
Hospital × group × surgery type	0.318	0.728
Side × group × surgery type	1.043	0.374

P: p value
